# Multimodal imaging in choroidal metastasis from primary adenocarcinoma of the esophagus: a case report

**DOI:** 10.22336/rjo.2021.52

**Published:** 2021

**Authors:** Fayna Rodríguez-González

**Affiliations:** * Ophthalmology Service, Hospital Universitario de Gran Canaria Doctor Negrín, Las Palmas de Gran Canaria, Spain

**Keywords:** choroidal metastasis, esophageal adenocarcinoma, ocular metastasis, photodynamic therapy

## Abstract

Adenocarcinoma of the esophagus is an extremely rare cause of choroidal metastasis. We report a case of a 53-year-old male with a six-month history of weight loss and two months of solid dysphagia, who presented with acute vision loss of his right eye, of 2 days of evolution. Ophthalmologic examination revealed bilateral choroidal masses associated with exudative retinal detachment in the right eye. Gastroduodenoscopy revealed a mass in the distal esophagus and endoscopic biopsy evidenced esophageal adenocarcinoma. The systemic study disclosed multiple pulmonary and liver metastatic nodules and enlarged thorax and abdomen lymph nodes. Systemic palliative chemotherapy was started, and specific ophthalmological treatment was ruled out given the patient’s situation. Choroidal metastasis of esophageal adenocarcinoma is a very rare cause of metastasis and there are very few cases reported until present in literature.

## Introduction

Metastatic carcinoma is the most common choroidal malignancy. Choroidal metastasis has been documented histologically in up to 12% of patients who die from cancer [**1**]. The most common primary tumor location is breast in women and lung in men. Conversely, choroidal metastasis from the gastrointestinal tract is much less common, 4% of cases approximately [**2**]. The most frequent gastrointestinal origins are colon, small intestine and stomach. The esophagus is an uncommon site of origin. Esophageal cancer presents two main histologic subtypes: squamous cell carcinoma and adenocarcinoma. Adenocarcinoma is much less frequent than squamous cell carcinoma. Thereby, while metastatic squamous cell carcinoma has been described more frequently, metastatic adenocarcinoma of the esophagus to the choroid has been reported in very few cases in literature [**3**-**8**]. We report a case of a man with choroidal metastasis from adenocarcinoma of the esophagus, who presented with acute vision loss in the right eye for 2 days long. 

## Case history

**Fig. 1:**
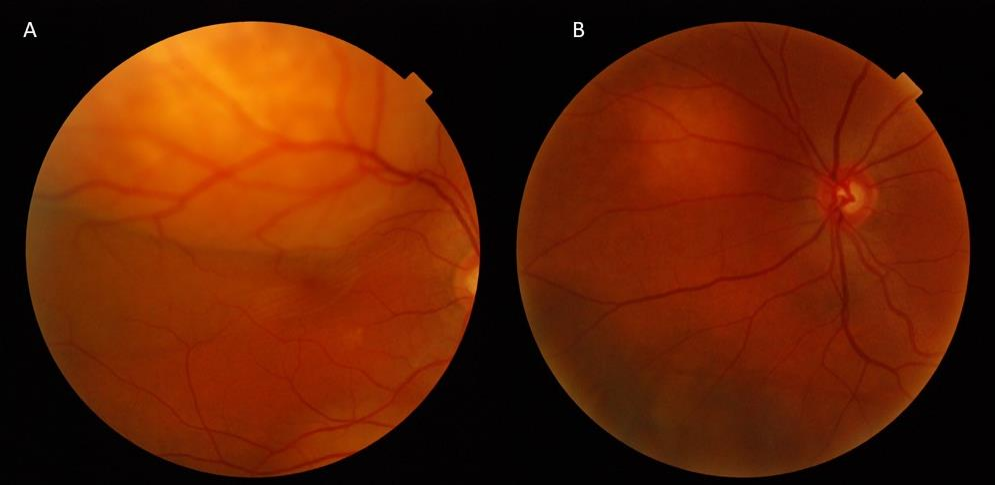
Right **(A)** and left eye **(B)** retinography at diagnosis. Choroidal lesions compatible with metastases, larger in the right eye

**Fig. 2:**
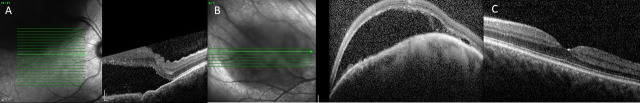
**A, B.** Optical coherence tomography at diagnosis. Large choroidal mass that produces neurosensory retinal detachment with macular involvement. **C.** Absence of macular involvement in the left eye

**Fig. 3:**
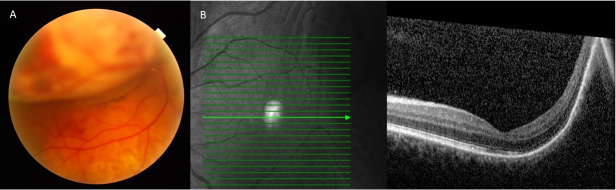
**A.** Right eye retinography showing an increase in the size of the lesions with greater involvement of the posterior pole coinciding with the clinical worsening. **B.** Left eye optical coherence tomography showing new onset macular involvement.

**Fig. 4:**
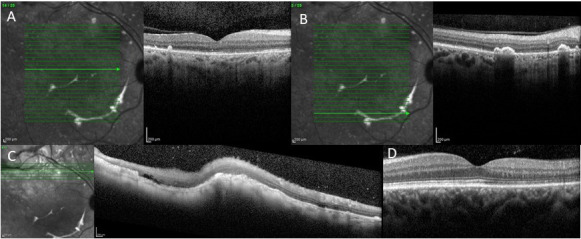
Optical coherence tomography of both eyes after chemotherapy treatment showing improvement of the lesions. **A, B, C.** Right eye. Central, superior and inferior section of the fovea respectively. **D.** Left eye

A 53-year-old man with recent onset of weight loss and solid dysphagia presented to the ophthalmology service with greater decreased vision in the left eye for 2 days. His personal history was significant for toxic grade chronic embolism, remarkable cigarette smoking and hepatitis C infection. Ophthalmologic examination revealed best-corrected visual acuity of 20/ 70 in the right eye and 20/ 20 in the left eye. Intraocular pressure and anterior segment examination were normal in both eyes. Funduscopic examination of the right eye revealed two yellow choroidal masses located in upper and lower temporal arcade respectively with associated exudative retinal detachment and macular involvement. Examination of the left eye revealed similar multiple choroidal lesions compatible with choroidal metastasis (**Fig. 1**, **2**). Ultrasonography demonstrated the choroidal origin of the lesions. Gastroduodenoscopy revealed a mass at 34 cm (distal esophagus) from the dental arch and endoscopic biopsy evidenced esophageal adenocarcinoma with gastroesophageal junction involvement. Computed tomography revealed multiple pulmonary and liver metastatic nodules and enlarged thorax and abdomen lymph nodes (stage IV esophageal adenocarcinoma). Systemic palliative chemotherapy with Carboplatin/ Paclitaxel was started at every three weeks, but no specific eye treatment was performed due to the advanced state of the disease and painless ocular lesions. After the start of chemotherapy, visual acuity, which had worsened until counting fingers in the right eye and 20/ 30 in the left eye (**Fig. 3**), slowly improved to a vision of 20/ 30 and 20/ 20 respectively after 6 chemotherapy sessions, while a regression in the size of the choroidal lesions was observed in both eyes (**Fig. 4**). Unfortunately, the patient died seven months after the diagnosis of the disease. 

## Discussion

Most of the metastatic tumors to the eye involve the uveal tract, whereas orbital and retinal metastases are extremely rare. Metastatic tumors usually include multifocal and bilateral lesions that can be associated with exudative retinal detachment and present with rapid growth and ultrasonographic features of medium-high internal reflectivity, in contrast to choroidal melanomas features with low internal reflectivity [**2**]. Breast and lung cancer represent more than 60% of the primary tumors’ location, whereas gastrointestinal tumors infrequently metastasize to the choroid, approximately 4% of cases [**2**,**9**]. At the time of ocular diagnosis, only two thirds of the patients had history of primary cancer, and one third of them had no previous history [**2**]. Metastasis from adenocarcinoma of the esophagus to the choroid has been reported in very few cases.

Adenocarcinoma of the esophagus is associated to Barrett esophagus, a condition in which distal esophageal squamous epithelium is replaced by columnar epithelium due to reflux of gastric juices. This entity is an aggressive malignancy; treatment strategies include surgical resection, radiotherapy and chemotherapy, but when distant metastases are present, as in our patient, the prognosis is poor. 

Moreover, adenocarcinoma of the esophagus is an exceedingly rare cause of choroidal metastasis, with only a few cases described previously in literature. However, an increasing incidence of this entity has been described recently, so that ophthalmologists should be aware of its metastatic potential to the eye, which has a clinical appearance similar to other metastatic lesions of different origin. Management of choroidal metastases depends on general patient status and include external beam radiation therapy, proton beam therapy and plaque brachytherapy [**2**]. Photodynamic therapy has been considered in some cases [**10**,**11**] and enucleation is reserved for the pain relief. In our case, no specific ocular treatment was preformed due to the advanced state of the illness and the absence of eye pain, only receiving systemic chemotherapy. It is worth highlighting the good ocular response to treatment, with significant improvement in visual acuity, which contributed to a better quality of life for the patient despite the poor general prognosis of the disease. 

## Conclusion

Choroidal metastasis from esophageal cancer is very rare, being esophageal adenocarcinoma much less frequent than squamous cell carcinoma. Ophthalmologists need to be aware of the metastatic potential of this disease, since, as in our case, the diagnosis can be suspected by ocular manifestations. Although systemic prognosis is poor, systemic palliative chemotherapy can improve visual acuity by partial regression of lesions, and, therefore, the patient’s quality of life. 

**Conflict of Interest statement**

Authors state no conflict of interest.

**Informed Consent and Human and Animal Rights statement**

Informed consent has been obtained from all individuals included in this study.

**Authorization for the use of human subjects**

Ethical approval: The research related to human use complies with all the relevant national regulations, institutional policies, is in accordance with the tenets of the Helsinki Declaration, and has been approved by the review board of Hospital Universitario de Gran Canaria Doctor Negrín, Las Palmas de Gran Canaria, Spain.

**Acknowledgements**

None.

**Sources of Funding**

None.

**Disclosures**

None.

## References

[1] Bloch RS, Gartner S (1971). The incidence of ocular metastatic carcinoma. Arch Ophthalmol.

[2] Shields CL, Shields JA, Gross NE, Schwartz GP, Lally SE (1997). Survey of 520 eyes with choroidal metastases. Ophthalmology.

[3] Samuel J, Flood TP, Agbemadzo B, Renta V, Mullai N, Osafo DC, Holloway N, Cohen JA (2003). Choroidal metastasis from adenocarcinoma of the esophagus. Retina.

[4] Singh D, Sharma A, Arora B, Shukla NK, Mohanti BK (2004). Adenocarcinoma esophagus with choroid metastasis. Indian J Gastroenterol.

[5] Knezevic J, Rodanovic N, Simic A, Radovanović AB, Bobić A, Latković Z, Pesko P (2003). Isolated choroidal metastasis from primary adenocarcinoma of the distal esophagus: a case report. Dis Esophagus.

[6] Buskens CJ, Tan HS, Hulscher JB, de Smet MD, van Lanschot JJ (2001). Adenocarcinoma of the esophagus with choroidal metastasis. Dis esophagus.

[7] Parikh HK, Deshpande RK, Swaroop DV, Desai PB (1993). Choroidal metastasis from primary adenocarcinoma of the esophagus. J Surg Oncol.

[8] Eliott D, Salehi-Had H, Plous OZ (2011). Adenocarcinoma of the esophagus presenting as choroidal metastasis. Dis Esophagus.

[9] Ferry AP, Font RL (1974). Carcinoma metastatic to the eye and orbit. I. A clinicopathologic study of 227 cases. Arch Ophthalmol.

[10] Isola V, Pece A, Pierro L (2006). Photodynamic therapy with verteporfin of choroidal malignancy from breast cáncer. Am J Ophthalmol.

[11] Harbour JW (2004). Photodynamic therapy for choroidal metastasis from carcinoid tumor. Am J Ophthalmol.

